# Machine learning-based classification of chronic traumatic brain injury using hybrid diffusion imaging

**DOI:** 10.3389/fnins.2023.1182509

**Published:** 2023-08-24

**Authors:** Jennifer J. Muller, Ruixuan Wang, Devon Milddleton, Mahdi Alizadeh, Ki Chang Kang, Ryan Hryczyk, George Zabrecky, Chloe Hriso, Emily Navarreto, Nancy Wintering, Anthony J. Bazzan, Chengyuan Wu, Daniel A. Monti, Xun Jiao, Qianhong Wu, Andrew B. Newberg, Feroze B. Mohamed

**Affiliations:** ^1^College of Engineering, Villanova University, Villanova, PA, United States; ^2^Department of Radiology, Thomas Jefferson University, Philadelphia, PA, United States; ^3^Marcus Institute of Integrative Health, Thomas Jefferson University, Philadelphia, PA, United States; ^4^Vickie and Jack Farber Institute for Neuroscience, Thomas Jefferson University, Philadelphia, PA, United States

**Keywords:** traumatic brain injury, machine learning, hybrid diffusion imaging, diffusion tensor imaging (DTI), neurite orientation dispersion and density imaging (NODDI)

## Abstract

**Background and purpose:**

Traumatic brain injury (TBI) can cause progressive neuropathology that leads to chronic impairments, creating a need for biomarkers to detect and monitor this condition to improve outcomes. This study aimed to analyze the ability of data-driven analysis of diffusion tensor imaging (DTI) and neurite orientation dispersion imaging (NODDI) to develop biomarkers to infer symptom severity and determine whether they outperform conventional T1-weighted imaging.

**Materials and methods:**

A machine learning-based model was developed using a dataset of hybrid diffusion imaging of patients with chronic traumatic brain injury. We first extracted the useful features from the hybrid diffusion imaging (HYDI) data and then used supervised learning algorithms to classify the outcome of TBI. We developed three models based on DTI, NODDI, and T1-weighted imaging, and we compared the accuracy results across different models.

**Results:**

Compared with the conventional T1-weighted imaging-based classification with an accuracy of 51.7–56.8%, our machine learning-based models achieved significantly better results with DTI-based models at 58.7–73.0% accuracy and NODDI with an accuracy of 64.0–72.3%.

**Conclusion:**

The machine learning-based feature selection and classification algorithm based on hybrid diffusion features significantly outperform conventional T1-weighted imaging. The results suggest that advanced algorithms can be developed for inferring symptoms of chronic brain injury using feature selection and diffusion-weighted imaging.

## Background and purpose

Traumatic brain injury (TBI) is a common condition with many potential acute and chronic neurological consequences (Smith et al., 2019), contributing to ~1 million deaths in the United States over the last two decades (Daugherty and Zhou, 2016). The neuropathology of chronic traumatic brain injury (cTBI) consists of a primary injury that is a direct consequence of traumatic insult, and a secondary injury that results from a cascade of molecular and cellular events including cell death, axonal injury, and inflammation (Anguita et al., 2022; Zhang et al., 2022). To better understand the underlying neuropathological mechanisms, there remains a growing need for information on the chronic effects of TBI (Wickwire et al., 2016).

Neuroimaging plays a critical role in the acute setting of brain injury, both in diagnosis and in guiding appropriate management by detecting injuries that require intervention or monitoring (Taylor and Gercel-Taylor, 2014; Douglas et al., 2015; Mckee and Daneshvar, 2015). However, in the setting of most mild-to-moderate injury, conventional T1-weighted imaging is typically normal (McCrory et al., 2009). Moreover, an initial assessment of TBI severity does not necessarily predict the extent of chronic disability (National Academies of Sciences, 2019). Therefore, advanced neuroimaging biomarkers are being actively researched in an attempt to better diagnose and monitor the acute and chronic effects of TBI (Hu et al., 2022).

Diffuse axonal injury is thought to be a key pathological mechanism underlying TBI and, as a result, it has led toward the development of advanced MR techniques for the visualization of WM integrity (Hashim et al., 2017). DTI and neurite orientation dispersion imaging (NODDI) (Zhang et al., 2012) are advanced MR techniques, which are thought to reflect the integrity of microstructural properties of the white matter (WM) in a range of clinical conditions. Diffusion tensor imaging (DTI) assumes Gaussian diffusion within a single microstructural compartment, whereas NODDI probes more complex, non-Gaussian properties using high-performance magnetic field gradients (Kamiya et al., 2020). Unlike DTI, NODDI uses seven parameters to measure the properties of the WM microstructure including intracellular, extracellular, and free water, whereas DTI is limited in its description of isotropic vs. anisotropic diffusion for a particular voxel (Muller et al., 2021).

Previously it has been shown that DTI and NODDI offer different, yet complementary, information regarding the microstructural integrity in patients with acute to chronic TBI (Wu et al., 2018; Palacios et al., 2020; Muller et al., 2021). Among DTI metrics, fractional anisotropy (FA) is the most studied and is often used as an indicator of white matter “integrity.” FA is mathematically defined as the square root of the sum of the squares of the eigenvalues within a diffusion tensor. Therefore, it is driven by axial diffusivity (AD; principle eigenvalue) and the second and third eigenvalues known as radial diffusivity (RD). DTI has been shown to be sensitive to acute WM changes, whereas NODDI has been shown to be more sensitive in detecting axonal degeneration over time (Timmers et al., 2016). Although DTI and NODDI may be more sensitive and specific for diagnosing TBI, they are rarely acquired in clinical practice, as advanced imaging techniques require additional scanning time, posing difficult challenges for practical application. To overcome this, a hybrid diffusion imaging (HYDI) sequence has been developed which acquires data that enable complementary post–data-processing strategies including DTI and NODDI (Wu and Alexander, 2007).

Machine learning (ML) methods have been demonstrated to be effective for various medical purposes (Chong et al., 2015; Vergara et al., 2017; Mohamed et al., 2022). The results of prior studies have suggested that biomarkers obtained from diffusion data, such as NODDI and DTI, combined with machine learning classification have the potential to be used for detecting neurodegenerative diseases such as Alzheimer's (Prasuhn et al., 2020) and Parkinson's (Pease et al., 2022) abnormalities. However, to the best of our knowledge, little research is conducted on using machine learning for inferring cognitive deficits in patients who experience cTBI (Qu et al., 2021). Currently, available algorithms lack the capabilities to make predictions in the absence of age-matched controls (Minaee et al., 2017) or fail to explore the diagnostic potential of higher-order diffusion models, such as NODDI (Qu et al., 2021). Notably, DTI and NODDI provide metrics with similar biological interpretations. These metrics are promising biomarkers for symptom development in TBI. Thus, the purpose of this study was to develop an ML model using various DTI and NODDI metrics fit from HYDI data, for inferring outcomes in patients with cTBI, and to compare its performance with conventional T1-weighted imaging.

## Materials and methods

This study was approved by the institutional review board of Thomas Jefferson University Hospital. All subjects included in the study provided informed consent.

### Subject cohort

A total of 59 subjects including 17 men and 42 women (age: 47 ± 15 years) experiencing chronic symptoms caused by a concussion-induced mild traumatic brain injury were recruited in this study. According to the Mayo Classification System for Traumatic Brain Injury Severity, mTBI was defined as a loss of consciousness of momentary for <30 min, amnesia for <24 h, with no positive MRI findings (Malec et al., 2007). A total of 22 of the 59 cTBI subjects had sustained a single concussion. Written informed consent, approved by the Institutional Review Board, was obtained from all subjects, and the study was registered on clinicaltrials.gov with the following identifier: NCT03241732. Subjects were recruited from the local community by self-referral and from local neurology offices and were excluded if they had a history of other neurological disorders, significant medical illness, a current substance-use disorder, or current *Diagnostic and Statistical Manual of Mental Disorders, 4th Edition* (DSM-IV) Axis I psychiatric illness. Subjects had to report a history of one or more prior TBIs with symptoms that lasted at least 3 months apart from the last concussion. All subjects had to meet criteria for mild traumatic brain injury including loss of consciousness <30 min, no significant amnesia, and no structural injury to the brain, such as hematoma, contusion, dura penetration, or brain stem injury. Symptoms had to emerge after the TBI and could include headache, hypersensitivity to auditory or visual stimuli, balance problems, cognitive problems, or emotional problems (i.e., depression or anxiety).

Clinical assessment of TBI subjects experiencing chronic symptoms included a battery of self-reported measures including the State-Trait Anxiety Inventory, Beck Depression Inventory, Profile of Mood Scale, Rivermead Post-Concussion Symptoms Questionnaire (RPQ-3 and RPQ-13), the Epworth Sleepiness Scale, and two cognitive tests, namely the forward and backward digit span and the Trails A and B test. Clinical assessments were performed on the same day as the imaging study. Subjects were classified as having favorable or unfavorable outcomes in each of the 21 tested neuropsychological outcomes, depending on whether their individual score was lower or higher than the mean value of the entire cohort. Patients with missing values or scores were not included in the final analysis. The characteristics of the full dataset are shown in Table 1.

**Table 1:** Characteristics of the 59 cTBI subjects.

**Demographics**	
No. of patients	59		
Age (mean+/-std) (yr)	46.8 +/– 15.3		
No. of male patients	17		
No. of female patients	42		
**Neuropsychological outcomes**	**All patients mean** ±**std**.	**Favorable outcome**	**Unfavorable outcome**
State anxiety	46.2 ± 15.4 (*n* = 57)	32.2 ± 10.5 (*n* = 31)	60.6 ± 9.0 (*n* = 26)
Trait anxiety	45.8 ± 14.1 (*n* = 57)	32.3 ± 10.7 (*n* = 29)	57.5 ± 9.3 (*n* = 28)
Beck depression inventory	17.9 ± 11.8 (*n* = 57)	8.6 ± 4.0 (*n* = 31)	28.4 ± 9.4 (*n* = 26)
Tension	13.5 ± 8.9 (*n* = 57)	6.8 ± 3.4 (*n* = 32)	22.0 ± 5.9 (*n* = 25)
Depression	14.3 ± 15.7 (*n* = 57)	4.0 ± 3.3 (*n* = 35)	32.0 ± 12.3 (*n* = 22)
Anger	10.4 ± 6.6 (*n* = 57)	4.1 ± 2.8 (*n* = 37)	19.6 ± 7.6 (*n* = 20)
Vigor	10.5 ± 6.6 (*n* = 57)	5.5 ± 2.9 (*n* = 30)	16.8 ± 4.0 (*n* = 27)
Fatigue	13.4 ± 7.4 (*n* = 57)	8.2 ± 3.5 (*n* = 31)	21.0 ± 4.3 (*n* = 26)
Confusion	12.4 ± 6.3 (*n* = 57)	7.7 ± 2.7 (*n* = 28)	18.1 ± 4.1 (*n* = 29)
Ability	16.1 ± 9.6 (*n* = 57)	9.3 ± 3.5 (*n* = 32)	25.6 ± 6.7 (*n* = 25)
Adjustment	18.8 ± 10.5 (*n* = 57)	10.2 ± 4.4 (*n* = 29)	27.7 ± 6.9 (*n* = 28)
Participation	9.1 ± 6.6 (*n* = 57)	4.5 ± 2.8 (*n* = 32)	15.4 ± 4.8 (*n* = 25)
May-portland	37.6 ± 20.9 (*n* = 57)	21.5 ± 7.7 (*n* = 31)	56.7 ± 14.5 (*n* = 26)
RPQ-3	5.5 ± 2.8 (*n* = 59)	3.3 ± 1.5 (*n* = 30)	7.9 ± 1.7 (*n* = 29)
RPQ-13	28.3 ± 11.0 (*n* = 59)	19.8 ± 7.5 (*n* = 28)	37.2 ± 6.0 (*n* = 31)
Headache	2.3 ± 1.3 (*n* = 59)	1.2 ± 0.8 (*n* = 18)	3.4 ± 0.5 (*n* = 41)
Forward digit span	10.4 ± 2.3 (*n* = 59)	8.6 ± 1.4 (*n* = 20)	12.2 ± 1.4 (*n* = 39)
Backward digit span	6.9 ± 2.3 (*n* = 59)	4.8 ± 1.0 (*n* = 27)	8.6 ± 1.4 (*n* = 32)
Epworth sleepiness scale	8.2 ± 5.1 (*n* = 59)	4.2 ± 2.1 (*n* = 29)	12.5 ± 2.9 (*n* = 30)
Trail making A (sec)	29.9 ± 15.2 (*n* = 59)	22.9 ± 4.1 (*n* = 40)	43.9 ± 19.1 (*n* = 19)
Trail making B (sec)	67.1 ± 27.4 (*n* = 59)	52.9 ± 8.7 (*n* = 38)	92.7 ± 31.0 (*n* = 21)

Several factors might affect the trajectory of disability and recovery after TBI, including age, sex, and type of injury. To account for these variables, all statistical analyses included age, sex, injury type, and imaging-to-injury time intervals as additional features to rule out associations.

### MR imaging protocol

*In vivo* brain data with HYDI were obtained on all chronic traumatic brain injury subjects using a 3T Siemens Biograph MR PET-MR scanner with a 32-channel head coil. For segmentation and registration of white matter atlas structures and to check whether or not any conventional positive radiological findings of brain injury could be detected, an anatomical T1-image was obtained for all cTBI and healthy control subjects. MRI parameters for the anatomical T1-weighted sequence were as follows: repetition time = 1.6 s, echo time = 2.46 ms, the field of view = 250 × 250 mm, matrix = 512 × 512, voxel size = 0.49 × 0.49 mm (Kraus et al., 2007), and 176 slices with slice thickness = 1 mm. The simultaneous multi-slice (SMS) HYDI pulse sequence was a single-shot, spin-echo, echo-planar imaging (SS-SE-EPI) pulse sequence with diffusion gradient pulses. The minimum b-value was 0 s/mm^2^ with five concentric diffusion-weighting shells (b-values = 250, 1,000, 2,000, 3,250, 4,000 s/mm^2^). A total of 144 diffusion-weighting gradient directions (6, 21, 24, 30, and 61 in each shell) were encoded. MRI parameters for the HYDI sequence were as follows: repetition time = 3.17 s, echo time = 120 ms, the field of view = 240 × 240 mm, matrix = 96 × 96, voxel size = 2.5 × 2.5 mm^2^, 63 slices with slice thickness = 2.5 mm, simultaneous multi-slice factor = 2, and total scan time of 8 min.

### Hybrid diffusion imaging preprocessing and atlas segmentation

**Figure 1:**
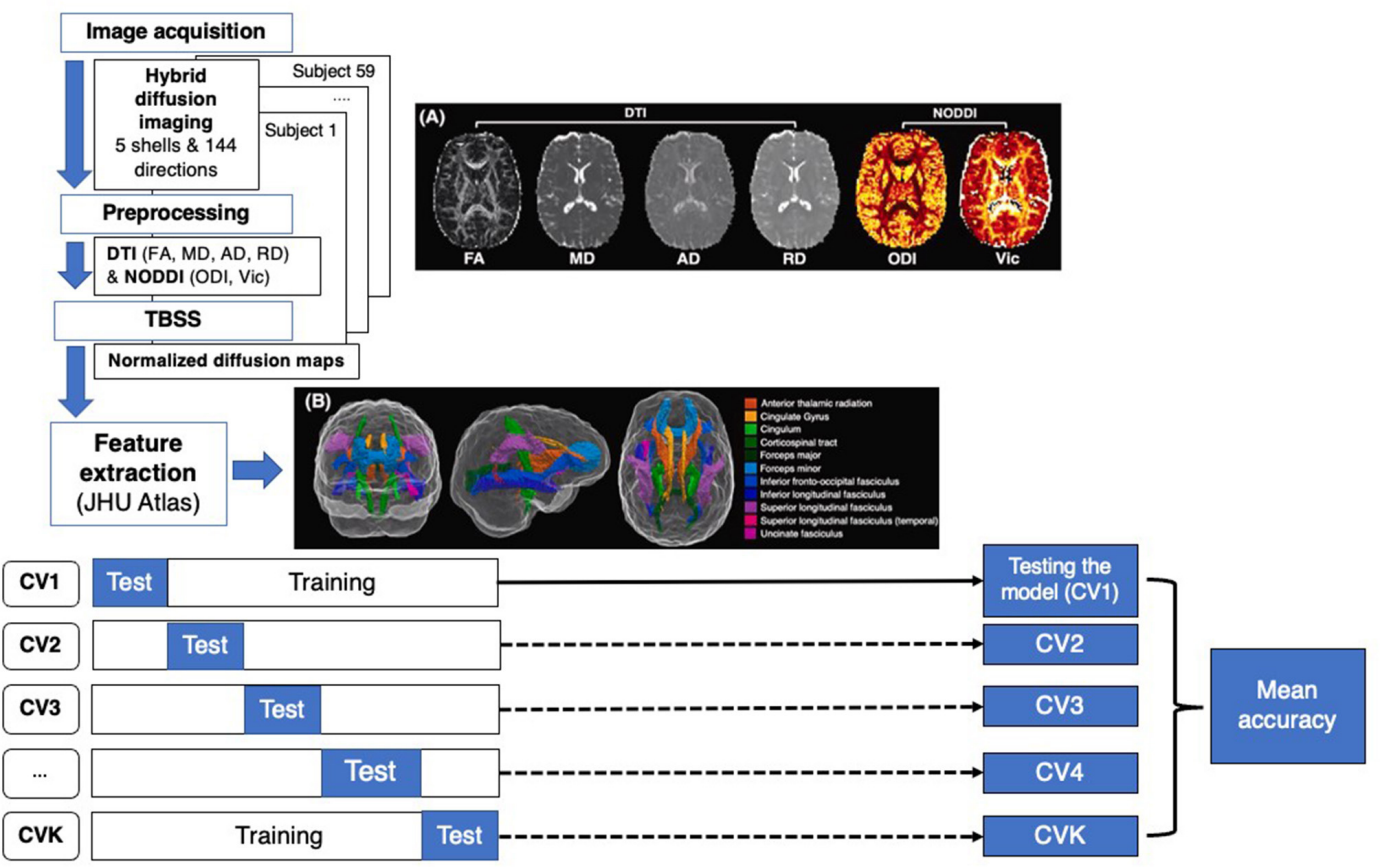
Flow chart representing machine learning (ML) approach for cTBI classification. Step 1 consists of feature extraction including image acquisition, preprocessing, image normalization and skeletonization using TBSS, and atlas registration. The dataset was divided into training and test datasets for K-fold CV, calculating the mean accuracy of each model.

Image processing included an initial preprocessing of the raw DICOM data and a computation of diffusion metrics. First, the susceptibility-induced distortion was estimated and corrected using the topup tool provided in the eddy current correction method of the FMRIB Software Library (FSL) (Jenkinson et al., 2012). The topup output was fed into the eddy tool by aligning all volumes to the b0 image. Prior to DTI and NODDI fitting, a denoising strategy was applied using MRtrix “dwidenoise,” which implements dMRI noise level estimation and denoising based on random matrix theory. DTI parameter maps were calculated similarly to a previous study from the inner two shells of the HYDI data using the FSL dtifit command and included FA, MD, AD, RD, and mean diffusivity (MD). Additionally, a MATLAB-based toolbox (https://www.nitrc.org/projects/noddi_toolbox) was used to compute higher-order diffusion metrics from the NODDI component of the analysis using the default model built into the NODDI toolbox. These included axonal density, also known as intra-cellular volume fraction (V_ic_), and the orientation dispersion index (ODI). Other parametric maps for NODDI were calculated but had no significant correlation with outcomes and were therefore not included in the final analysis. Diffusion and T1-weighted images were aligned to a common template in Montreal Neurological Institute152 (MNI152) standard space, using a non-linear registration algorithm FNIRT (part of FSL) (Figure 1). Region-based metrics were calculated for each subject by averaging the diffusion and T1 metrics within each of the 20 regions from the Johns Hopkins University white matter tractography atlas mapped onto the standard MNI152 space (Figure 1).

### Development of the machine learning-based classification model

We utilized the scikit-learn library, a versatile library for ML models and operations based on Python, to build multiple ML classification models. In this experiment, we developed five different ML models based on different algorithms including support vector machine (SVM) (Pontil and Verri, 1998; Suthaharan, 2016), K-nearest neighbors (K-NNs), logistic regression (LR), random forest (RF) (Rodriguez-Galiano et al., 2012; Palczewska et al., 2014), and decision tree (DT) (Myles et al., 2004; Grabmeier and Lambe, 2007). SVM is a supervised learning method searching for an optimal hyperplane separation between classes, which maximizes the classification margin. LR is a statistical technique used to infer the relationship between dependent and independent variables. K-NN tries to infer the correct class for the test data by calculating the distance between the test data and training points. RF is an ML algorithm based on the idea of creating a highly accurate inference rule by boosting or bragging many relatively weak and inaccurate rules to improve a single estimator (Jost, 2006; Langs et al., 2011). For all ML methods, the default parameters provided by the scikit-learn library were used (https://scikit-learn.org/stable/) (Kramer, 2016). Details of the different classification algorithms are provided in Appendix B. We trained and tested all T1, DTI (FA, AD, MD, RD), and NODDI (ODI, Vic) metrics separately using all five ML algorithms for a total of 35 inferences.

### Feature extraction and ranking

Before training ML models, it is important to extract useful features based on feature engineering. Extraction of an important set of features not only improves the inference accuracy but also would reduce the overfitting possibility (Musavi et al., 1992; Rätsch et al., 2007; Mutasa et al., 2020; Rafało, 2022). Using the same T1, DTI, and NODDI features, a proposed ML-based classification pipeline was developed, which consisted of a feature ranking module followed by a classification model. In this study, we selected the DT and K-NN models for feature selection and classification model, respectively. The DT module ranked the 20 different brain regions by calculating the number of samples that reach a particular feature (T1, FA, AD, MD, RD, ODI, and Vic), divided by the total number of samples. The higher the value, the more important the feature. Then the top six features were selected to be fed into the classifier to classify the extent of cognitive impairment as measured by the trail-making task (details in Appendix C). We select K-NN as the classifier since it considers the distance between different data samples to treat each feature with equal weights (Hu et al., 2016). To this end, we compare the performance of K-NN to illustrate the performance of feature selection.

### Evaluation of ML models

Similar to other studies applying ML to the medical domain (Lao et al., 2004; Chong et al., 2015; Razzak et al., 2018; Davatzikos, 2019), we have a limited dataset. Thus, to improve the data utilization and reduce the possibility of overfitting, we used a K-fold cross-validation (CV) (Refaeilzadeh et al., 2016; Mutasa et al., 2020; Anguita et al., 2022) approach to evaluate the performance of our ML models. In particular, we used the stratified cross-validator “StratifiedKFold” of the scikit-learn tool in the evaluations, which enabled us to compare the classification performance based on the same conditions. In general, the dataset was split into a training set and a testing set. Employing a K-fold CV, the dataset was first randomly divided into K equal-size subsets. CV1, CV2… …CVK represents the CV iteration 1, 2… ….K, with K representing the total number of iterations. Then the K subsets are iteratively selected as the testing set, and the training set consists of the remaining data (see Appendix A). In our experiment, without loss of generality, we selected *K* = 10, which means the classification performance was evaluated and averaged over 10 rounds of CV. The classification models were trained using only the training set, and performance was evaluated using the testing set.

## Results

**Figure 2:**
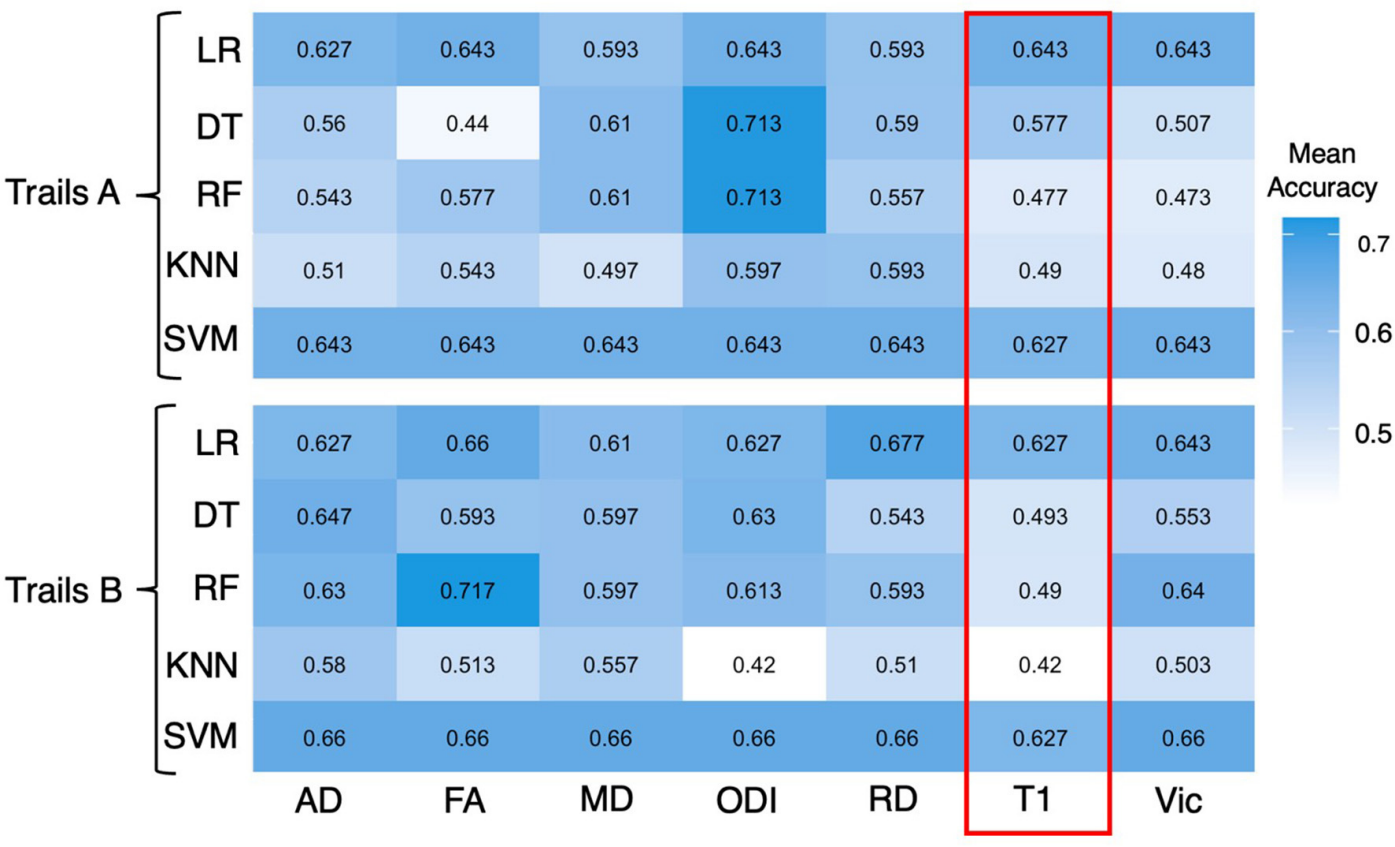
Heatmap showing mean accuracy performance of different ML algorithms for a trail making A and B. All 20 JHU atlas regions are used as features for the above figure. Mean accuracy results based on T1 inferences are highlighted in red and are expressed in percentages.

**Figure 3:**
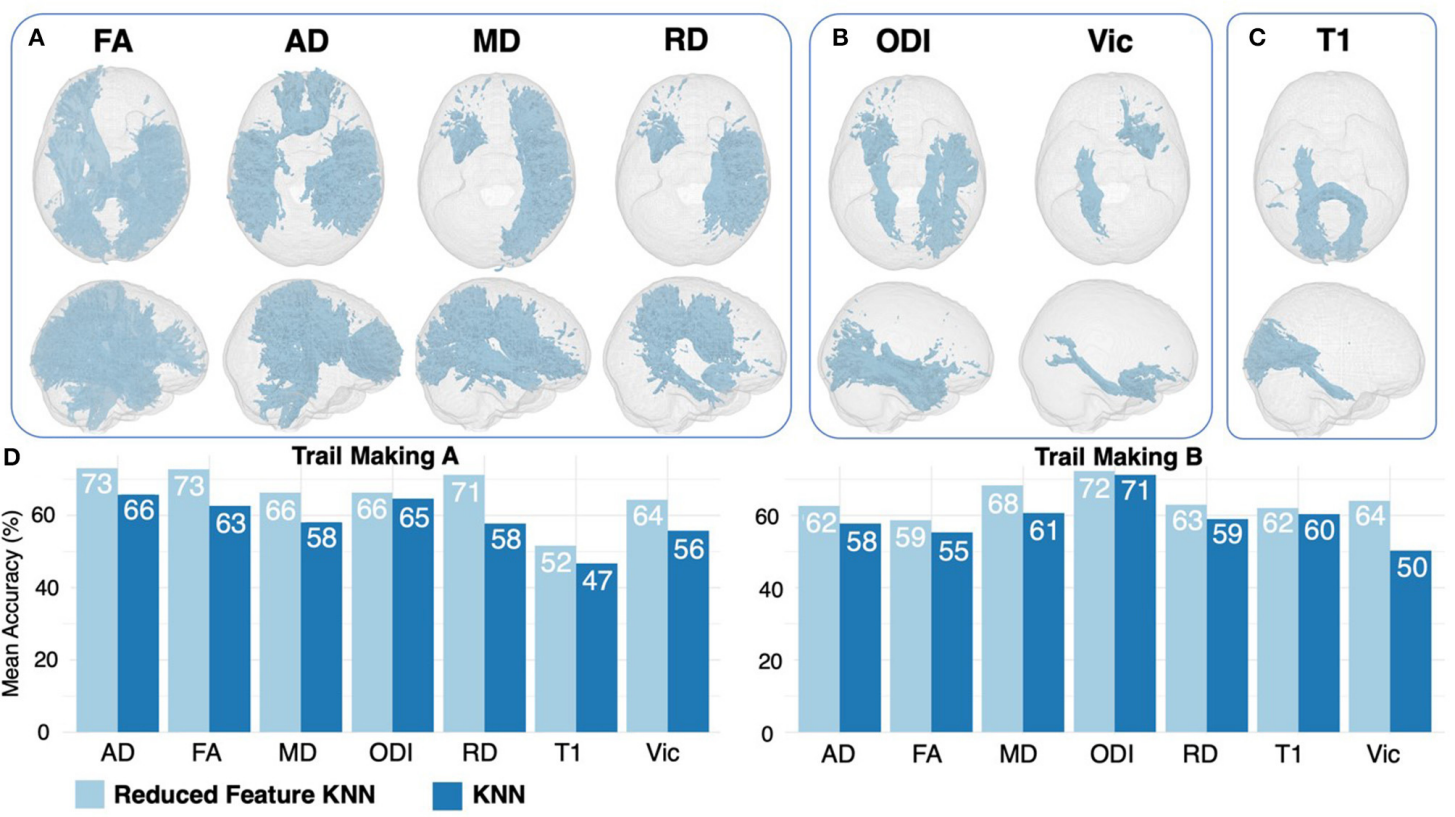
Feature ranking results for DTI **(A)**, NODDI **(B)**, and T1 **(C)** regions. Features are displayed if they were ranked as significant for both trail making A and B. Results of 6-feature KNN are displayed in light blue, compared with 20-feature KNN results in dark blue **(D)**.

As the trail-making task has been shown to be a reliable indicator of potential signs of cognitive impairment, we used the time it took for patients to complete trail making A and B (seconds) to evaluate the diagnostic potential of the machine learning algorithms. A longer time to complete trail making indicates worse performance for this task. Age, sex, number of TBI, and injury-to-imaging interval did not have a significant prediction on the classification of symptoms, indicating no significant correlation or confounding effects. The experimental results are indicated in Figures 2, 3.

### Diagnostic performance of DTI and NODDI vs. T1-weighted imaging

Across ML algorithms, the mean accuracy for T1, FA, AD, MD, RD, Vic, and ODI as expressed in percentages was 0.551 (0.517–0.568), 0.61 (0.440–0.727), 0.615 (0.510–0.730), 0.610 (0.497–0.683), 0.610 (0.510–0.713), 0.590 (0.473–0.660), and 0.637 (0.420–0.723), respectively (Table 2). The individual mean accuracy for each diffusion metric for Trails A and B can be observed in the heatmap within Figure 2. T1 features were significantly less accurate in predicting cognitive performance in patients with cTBI compared with diffusion metrics (*P* < 0.05) and NODDI (*P* < 0.05). ODI exhibited the highest mean accuracy of all of the features tested. When using all 20 features, the mean accuracy of logistic regression, decision tree, random forest, k-nearest neighbors, and support vector machine was 0.648 (0.510–0.677), 0.515 (0.420–0.597), 0.589 (0.473–0.713), 0.575 (0.440–0.717), and 0.633 (0.593–0.647), respectively. When considering all 20 features, the logistic regression method was the most predictive of cTBI classification, with SVM being the second most accurate, having the highest predictive accuracy when averaged across DTI metrics (0.633 and 0.648, respectively), showing the robustness and predictive ability for these two algorithms.

**Table 2:** Diagnostic performance across all ML algorithms, including LR, DT, RF, K-NN, SVM, and combined feature selection with K-NN.

**Metric** **(% accuracy range)**	**Mean accuracy (range) (average) (range)**	*P*-value^*^
T1 (51.7–56.8%)	55.1% (51.7–56.8%)	–
**DTI (58.7–73.0%)**		
FA	61.0% (44.0–72.7%)	0.030
AD	61.5% (51.0–73.0%)	0.009
MD	61.0% (49.7–68.3%)	0.005
RD	61.0% (51.0–71.3%)	0.004
**NODDI (64.0–72.3%)**		
Vic	59.0% (47.3–66.0%)	0.036
ODI	63.7% (42.0–72.3%)	0.001

^*^P-values: between T1 and DTI or NODDI metric.

### Diagnostic performance of combined decision tree ranking and feature importance

Feature ranking using the DT method returned the top 33% of the most important features based on variable importance (Table 3). When comparing results between trail making A and B, several overlapping features were ranked as important across all DTI, NODDI, and T1 maps (Table 3, Figures 3A–C). By feeding the selected features to the K-NN model, we improved the mean accuracy of inference by ~11.4% (*P* < 0.001).

**Table 3:** Feature ranking results for each test and metric using the DT algorithm.

**Metric**	**Test**	**Selected features**
T1	Trail Making A	Cingulum (cingulate gyrus) L, Cingulum (cingulate gyrus) R, Cingulum (hippocampus) L, Forceps major, Inferior fronto-occipital fasciculus L, Superior longitudinal fasciculus (temporal part) L
T1	Trail Making B	Corticospinal tract L, Cingulum (hippocampus) L, Forceps major, Superior longitudinal fasciculus R, Superior longitudinal fasciculus (temporal part) L, Superior longitudinal fasciculus (temporal part) R
**DTI**		
FA	Trail Making A	Anterior thalamic radiation L, Corticospinal tract R, Forceps major, Inferior longitudinal fasciculus L, Inferior longitudinal fasciculus R, Superior longitudinal fasciculus R
FA	Trail Making B	Anterior thalamic radiation L, Corticospinal tract R, Forceps major, Inferior longitudinal fasciculus R, Superior longitudinal fasciculus R, Superior longitudinal fasciculus (temporal part) R
AD	Trail Making A	Corticospinal tract R, Forceps major, Forceps minor, Superior longitudinal fasciculus L, Superior longitudinal fasciculus R, Superior longitudinal fasciculus (temporal part) L
AD	Trail Making B	Corticospinal tract R, Forceps minor, Superior longitudinal fasciculus L, Superior longitudinal fasciculus R, Uncinate fasciculus R, Superior longitudinal fasciculus (temporal part) L
MD	Trail Making A	Forceps major, Inferior fronto-occipital fasciculus R, Superior longitudinal fasciculus L, Superior longitudinal fasciculus R, Uncinate fasciculus L, Superior longitudinal fasciculus (temporal part) R
MD	Trail Making B	Corticospinal tract R, Forceps minor, Inferior fronto-occipital fasciculus R, Superior longitudinal fasciculus R, Uncinate fasciculus L, Uncinate fasciculus R
RD	Trail Making A	Cingulum (cingulate gyrus) L, Cingulum (hippocampus) R, Inferior fronto-occipital fasciculus R, Superior longitudinal fasciculus L, Superior longitudinal fasciculus R, Uncinate fasciculus L
RD	Trail Making B	Anterior thalamic radiation L, Cingulum (hippocampus) R, Superior longitudinal fasciculus R, Uncinate fasciculus L, Uncinate fasciculus R, Superior longitudinal fasciculus (temporal part) L
**NODDI**		
ODI	Trail Making A	Anterior thalamic radiation L, Cingulum (hippocampus) L, Cingulum (hippocampus) R, Inferior longitudinal fasciculus R, Uncinate fasciculus L, Superior longitudinal fasciculus (temporal part) R
ODI	Trail Making B	Corticospinal tract L, Cingulum (hippocampus) L, Cingulum (hippocampus) R, Inferior longitudinal fasciculus R, Superior longitudinal fasciculus R, Uncinate fasciculus L
Vic	Trail Making A	Cingulum (hippocampus) L, Inferior longitudinal fasciculus L, Superior longitudinal fasciculus R, Uncinate fasciculus L, Uncinate fasciculus R, Superior longitudinal fasciculus (temporal part) L
Vic	Trail Making B	Inferior longitudinal fasciculus L, Corticospinal tract L, Cingulum (hippocampus) L, Forceps major, Inferior longitudinal fasciculus R, Uncinate fasciculus R

## Discussion

To the best of our knowledge, this is the first study to describe the development of an ML-based classification algorithm to compare DTI and NODDI, in a real clinical setting by analyzing the same data, for the diagnosis of cTBI. In clinical practice, it is difficult to infer symptoms of TBI using conventional MR imaging, though several studies using DTI and NODDI have been shown to correlate with symptoms (Palacios et al., 2020). MR imaging findings in cTBI are often subtle, with DTI and NODDI rarely being acquired, and visual assessments of these findings being subjective (Zhang et al., 2012). Moreover, ML models have not yet been widely developed and verified within the field of diagnosis, creating a need for ML methods to infer symptom pathology from neuroimaging, particularly within a real-world clinical setting. We found that DTI and NODDI resulted in mean accuracies of 58.7–73.0% and 64.0–72.3%, respectively, and consistently outperformed T1-weighted imaging in all ML algorithms across multiple testing methods to infer symptoms of cognitive impairment. Our results are informative for the community and elucidate whether NODDI and DTI detect changes in similar locations, are more predictive than conventional T1-weighted imaging, and whether there are advantages to acquiring an advanced diffusion sequence for the inference of cTBI.

The DT feature-reduction method developed in this study successfully improved the performance of K-NN, with an 11% mean improvement in inference accuracy. This suggests that the effects of cTBI may be more localized to specific brain regions, rather than widespread throughout the whole brain. The results of the DT feature ranking method improving the predictive accuracy suggests that clinicians should focus on specific regions when looking for signs of clinical impairment as more specific areas of the brain appear to be correlated with clinical outcomes. Our data are generally consistent with previous findings, where importance-based variable selection has been shown to allow for simplified predictive models to be created while maintaining inference accuracy. In a diverse unpredictable condition, such as cTBI, these results help refine our approach to head injury assessment, decision-making, and outcome inference targeted at model sensitivity and specificity.

This study has several limitations. First, this study was based on a relatively small subject cohort for the development of an ML algorithm. A larger subject cohort including a wider range of outcomes from multiple centers is desirable to increase the generalizability and robustness of the algorithm. This study, however, was not intended to develop an all-inclusive tool to differentiate various causes of cognitive impairment but rather to compare the predictive ability of DTI, NODDI, and T1-weighted imaging. The HYDI data collected were done so in a timeframe that may be clinically feasible for most clinical centers. Further validation with larger, prospectively collected test datasets may be necessary to determine whether this method is applicable to various types of cognitive impairment. Second, diagnostic criteria for cTBI were based on clinical diagnosis and self-reported symptoms; this is not only a weakness but also a strength as this is more generalizable to what would be experienced, given a real-world clinical setting. However, more robust inferences could be generated by using more quantitative measurements of cognitive abilities.

Finally, our preliminary experiment selects feature engineering-based approaches and utilizes DT for feature ranking and traditional ML for testing. In future studies, we will consider more sophisticated feature ranking methods, such as Lasso regression, and more powerful classifiers including multilayer perceptron (MLP) and graph neural networks (GNNs).

The clinical applications of the development of our ML models are extensive, as it not only indicates that deficits in cognition can be predicted in cTBI patients in the absence of age-and-sex-matched healthy controls but also highlights the utility of adopting advanced diffusion imaging into a clinical pipeline of what is traditionally used. Our findings could affect not only the diagnosis and treatment monitoring of patients with cTBI but also could offer a method of determining a patient's likelihood of developing cognitive deficits within the acute stages of injury.

## Conclusion

ML-based classification algorithms using DTI and NODDI consistently outperform conventional T1-weighted brain imaging for predicant patients' symptoms and unfavorable outcomes 6 months following the traumatic incident. Reducing the number of features improves the accuracy of inference. These results indicate that ML-based classification built on higher-order diffusion modeling and reduced features may be a promising tool for inferring cognitive deficits in TBI, improving clinical decision-making and yielding better patient outcomes.

## Data availability statement

The original contributions presented in the study are included in the article/supplementary material, further inquiries can be directed to the corresponding author.

## Ethics statement

The studies involving human participants were reviewed and approved by IRB. Written informed consent to participate in this study was provided by the participants' legal guardian/next of kin.

## Author contributions

JM and RW: conceptualization, methodology, investigation, software, validation, and writing-original draft preparation. DMi and MA: conceptualization, data curation, and software. KK and RH: data curation, formal analysis, and investigation. GZ, NW, CW, DMo, and AB: resources, supervision, and project administration. CH and EN: resources, data curation, and project administration. XJ: conceptualization, methodology, formal analysis, investigation, software, resources, and writing - review and editing. QW: conceptualization, methodology, investigation, resources, supervision, and writing - review and editing. AN: conceptualization, methodology, investigation, software, resources, supervision, project administration, and writing - review and editing. FM: conceptualization, methodology, investigation, formal analysis, investigation, software, resources, project administration, supervision, and writing - review and editing.

## References

[B1] Anguita D. Ghelardoni L. Ghio A. Oneto L. Ridella S. (2022). The ‘K' in K-fold Cross Validation, Eds G. Balint, Antala B, Carty C, Mabieme JMA, Amar IB, Kaplanova A (Uniwersytet ślaski), 441–446.

[B2] Chong S. L. Liu N. Barbier S. Ong M. E. H. (2015). Predictive modeling in pediatric traumatic brain injury using machine learning data analysis, statistics and modelling. BMC Med. Res. Methodol. 15, 1–9. 10.1186/s12874-015-0015-0 25886156 PMC4374377

[B3] Daugherty J. Zhou H. Sarmiento K. Waltzman D. (2016). Differences in state traumatic brain injury-related deaths, by principal mechanism of injury, intent, and percentage of population living in rural areas-United States, 2016-2018. MMWR. 15. 34648483 10.15585/mmwr.mm7041a3 PMC8631284

[B4] Davatzikos C. (2019). Machine learning in neuroimaging: progress and challenges. Neuroimage. 197, 652. 10.1016/j.neuroimage.2018.10.003 30296563 PMC6499712

[B5] Douglas D. B. Iv M. Douglas P. K. Vos S. B. Bammer R. Zeineh M.. (2015). Diffusion tensor imaging of TBI: potentials and challenges HHS public access. Top. Magn. Reson. Imaging. 24, 241–251. 10.1097/RMR.0000000000000062 26502306 PMC6082670

[B6] Grabmeier J. L. Lambe L. A. (2007). Decision trees for binary classification variables grow equally with the Gini impurity measure and Pearson's chi-square testInt. J. Bus. Intell. Data Min. 2, 213–226. 10.1504/IJBIDM.2007.013938

[B7] Hashim E. Caverzasi E. Papinutto N. Lewis C. E. Jing R. Charles O.. (2017). Investigating microstructural abnormalities and neurocognition in sub-acute and chronic traumatic brain injury patients with normal-appearing white matter: a preliminary diffusion tensor imaging study. Front. Neurol. 8, 97. 10.3389/fneur.2017.00097 28373856 PMC5357974

[B8] Hu L. Yang S. Jin B. Wang C. (2022). Advanced neuroimaging role in traumatic brain injury: a narrative review. Front. Neurosci. 16, 453. 10.3389/fnins.2022.872609 35495065 PMC9043279

[B9] Hu L. Y. Huang M. W. Ke S. W. Tsai C. F. (2016). The distance function effect on k-nearest neighbor classification for medical datasets. Springerplus. 5, 1–9. 10.1186/s40064-016-2941-7 27547678 PMC4978658

[B10] Jenkinson M. Beckmann C. F. Behrens T. E. J. Woolrich M. W. Smith S. M. (2012). Review FSL. Neuroimage. 62, 782–790. 10.1016/j.neuroimage.2011.09.015 21979382

[B11] Jost L. (2006). Entropy and diversity. Oikos. 113, 363–375. 10.1111/j.2006.0030-1299.14714.x

[B12] Kamiya K. Hori M. Aoki S. (2020). NODDI in clinical research. J. Neurosci. Methods. 346, 108908. 10.1016/j.jneumeth.2020.108908 32814118

[B13] Kramer O. (2016). “Scikit-Learn” in Machine Learning for Evolution Strategies, 45–53. 10.1007/978-3-319-33383-0_5

[B14] Kraus M. F. Susmaras T. Caughlin B. P. Walker C. J. Sweeney J. A. Little D. M.. (2007). White matter integrity and cognition in chronic traumatic brain injury: a diffusion tensor imaging study. Brain. 130, 2508–2519. 10.1093/brain/awm216 17872928

[B15] Langs G. Menze B. H. Lashkari D. Golland P. (2011). Detecting stable distributed patterns of brain activation using Gini CONTRAST. Neuroimage. 56, 497–507. 10.1016/j.neuroimage.2010.07.074 20709176 PMC3960973

[B16] Lao Z. Shen D. Xue Z. Karacali B. Resnick S. M. Davatzikos C.. (2004). Morphological classification of brains via high-dimensional shape transformations and machine learning methods. Neuroimage. 21, 46–57. 10.1016/j.neuroimage.2003.09.027 14741641

[B17] Malec J. F. Brown A. W. Leibson C. L. Flaada J. T. Mandrekar J. N. Diehl N. N.. (2007). The mayo classification system for traumatic brain injury severity. J. Neurotrauma. 24, 1417–1424. 17892404 10.1089/neu.2006.0245

[B18] McCrory P. Meeuwisse W. Johnston K. Dvorak J. Aubry M. Molloy M.. (2009). Consensus statement on concussion in sport: the 3rd international conference on concussion in sport held in Zurich, november 2008. Br. J. Sports Med. 43, i76–i84. 10.1136/bjsm.2009.058248 19433429

[B19] Mckee A. C. Daneshvar D. H. (2015). The neuropathology of traumatic brain injury. Handb. Clin. Neurol. 127, 45–66. 25702209 10.1016/B978-0-444-52892-6.00004-0 PMC4694720

[B20] Minaee S. Wang Y. Chung S. Wang X. Fieremans E. Flanagan S.. (2017). A machine learning approach for identifying patients with mild traumatic brain injury using diffusion MRI modeling. arXiv Preprint. arXiv:1708.09000.

[B21] Mohamed M. Mohamed M. Khalid N. (2022). Prognosticating outcome using magnetic resonance imaging in patients with moderate to severe traumatic brain injury: a machine learning approach. 10.1080/02699052.2022.2034184 35129403

[B22] Muller J. Middleton D. Alizadeh M. Zabrecky G. Wintering N. A. Bazzan A. J.. (2021). Hybrid diffusion imaging reveals altered white matter tract integrity and associations with symptoms and cognitive dysfunction in chronic traumatic brain injury. Neuroimage. Clin. 30, 102681. 10.1016/j.nicl.2021.102681 34215151 PMC8102667

[B23] Musavi M. T. Ahmed W. Chan K. H. Faris K. B. Hummels D. M. (1992). On the training of radial basis function classifiers. Neural. Networks. 5, 595–603. 10.1016/S0893-6080(05)80038-3

[B24] Mutasa S. Sun S. Ha R. (2020). Understanding artificial intelligence based radiology studies: what is overfitting? Clin. Imaging. 65, 96–99. 10.1016/j.clinimag.2020.04.025 32387803 PMC8150901

[B25] Myles A. J. Feudale R. N. Liu Y. Woody N. A. Brown S. D. (2004). An introduction to decision tree modeling. J. Chemom. 18, 275–285. 10.1002/cem.873

[B26] National Academies of Sciences Engineering, and Medicine; Health and Medicine Division; Board on Health Care Services; Committee on the Review of the Department of Veterans Affairs Examinations for Traumatic Brain Injury. (2019). Evaluation of the Disability Determination Process for Traumatic Brain Injury in Veterans. Washington (DC): National Academies Press. 31211534

[B27] Palacios E. M. Owen J. P. Yuh E. L. Vassar M. J. Ferguson A. R. Diaz-arrastia R.. (2020). The evolution of white matter microstructural changes after mild traumatic brain injury: a longitudinal DTI and NODDI study. Sci. Adv. 6, aaz6892. 10.1126/sciadv.aaz6892 32821816 PMC7413733

[B28] Palczewska A. Palczewski J. Robinson R. M. Neagu D. (2014). Interpreting random forest classification models using a feature contribution method. Adv. Intell. Syst. Comput. 263, 193–218. 10.1007/978-3-319-04717-1_9

[B29] Pease M. Arefan D. Barber J. Yuh E. Puccio A. Hochberger K.. (2022). Outcome prediction in patients with severe traumatic brain injury using deep learning from head CT scans. Radiology. 304, 385–394. 35471108 10.1148/radiol.212181 PMC9340242

[B30] Pontil M. Verri A. (1998). Properties of support vector machines. Neural. Comput. 10, 955–974. 10.1162/089976698300017575 9573414

[B31] Prasuhn J. Heldmann M. Münte T. F. Brüggemann N. (2020). A machine learning-based classification approach on Parkinson's disease diffusion tensor imaging datasets. Neurol. Res. Pract. 2, 1–5. 10.1186/s42466-020-00092-y 33324945 PMC7654034

[B32] Qu Y. Wang P. Liu B. Song C. Wang D. Yang H.. (2021). AI4AD: Artificial intelligence analysis for Alzheimer's disease classification based on a multisite DTI database. Brain Disord. 1, 100005. 10.1016/j.dscb.2021.100005

[B33] Rafało M. (2022). Cross validation methods: analysis based on diagnostics of thyroid cancer metastasis. ICT Express. 8, 183–188. 10.1016/j.icte.2021.05.001

[B34] Rätsch G. Warmuth M. K. K. Glocer K. (2007). Boosting algorithms for maximizing the soft margin. Adv Neural Inf Process Syst. 20.20975119

[B35] Razzak M. I. Naz S. Zaib A. (2018). Deep learning for medical image processing: overview, challenges and the future BT-classification in BioApps: automation of decision making. Springer. 26, 323–350. 10.1007/978-3-319-65981-7_12

[B36] Refaeilzadeh P. Tang L. Liu H. (2016). “Cross-validation” in Encyclopedia of Database Systems (New York, NY: Springer New York), 1–7. 10.1007/978-1-4899-7993-3_565-2

[B37] Rodriguez-Galiano V. F. Ghimire B. Rogan J. Chica-Olmo M. Rigol-Sanchez J. P. (2012). An assessment of the effectiveness of a random forest classifier for land-cover classification. ISPRS J. Photogramm. Remote Sens. 67, 93–104. 10.1016/j.isprsjprs.2011.11.002

[B38] Smith L. G. F. Milliron E. Ho M. L. Hu HH Rusin J Leonard J. (2019). Advanced neuroimaging in traumatic brain injury: an overview. Neurosurg. Focus. 47, E17. 10.3171/2019.9.FOCUS19652 32364704

[B39] Suthaharan S. (2016). “Support vector machine.” in Machine Learning Models and Algorithms for Big Data Classification, 207–235. 10.1007/978-1-4899-7641-3_9

[B40] Taylor D. D. Gercel-Taylor C. (2014). Exosome platform for diagnosis and monitoring of traumatic brain injury. Philos. Trans. R. Soc. B: Biol. Sci. 369, 20130503. 25135964 10.1098/rstb.2013.0503 PMC4142024

[B41] Timmers I. Roebroeck A. Bastiani M. Jansma B. Rubio-Gozalbo E. Zhang H.. (2016). Assessing microstructural substrates of white matter abnormalities: A Comparative study using DTI and NODDI. PLoS ONE. 11, 1–15. 10.1371/journal.pone.0167884 28002426 PMC5176300

[B42] Vergara V. M. Mayer A. R Kiehl K. A. (2017). Detection of mild traumatic brain injury by machine learning classification using resting state functional network connectivity and fractional anisotropy. J. Neurotrauma. 27676221 10.1089/neu.2016.4526 PMC5333571

[B43] Wickwire E. M. Williams S. G. Roth T. Capaldi V. F. Jaffe M. Moline M.. (2016). Sleep, sleep disorders, and mild traumatic brain injury. What we know and what we need to know: findings from a national working group. Neurotherapeutics. 13, 403–417. 10.1007/s13311-016-0429-3 27002812 PMC4824019

[B44] Wu Y. C. Alexander A. L. (2007). Hybrid diffusion imaging. Neuroimage. 36, 617. 10.1016/j.neuroimage.2007.02.050 17481920 PMC2428345

[B45] Wu Y. C. Mustafi S. M. Harezlak J. Kodiweera C. Flashman L. A. Mcallister T. W.. (2018). Hybrid diffusion imaging in mild traumatic brain injury. J. Neurotrauma. 35, 2377–2390. 10.1089/neu.2017.5566 29786463 PMC6196746

[B46] Zhang H. Schneider T. Wheeler-Kingshott C. A. Alexander D. C. (2012). NODDI practical in vivo neurite orientation dispersion and density imaging of the human brain. Neuroimage. 61, 1000–1016. 10.1016/j.neuroimage.2012.03.072 22484410

[B47] Zhang T. Du C. Wang J. (2022). Composite Quantization for Approximate Nearest Neighbor Search, 838–846.Available online at: https://proceedings.mlr.press/v32/zhangd14.html (accessed August 31, 2022).

