# Supplementary material for: Machine learning-based classification of chronic traumatic brain injury using hybrid diffusion imaging
Source: Front Neurosci. 2023 Aug 24;17:1182509. doi: 10.3389/fnins.2023.1182509 (PMC10484001; doi:10.3389/fnins.2023.1182509)
Supplement: Supplementary file 1 [file Data_Sheet_1.docx]

**Appendix A: K-Fold Cross Validation**

Training ML models on an entire dataset result in the model outperforming on the test set, and over-fitting. If all the data including features and labels were already seen by the model, the model creates a much higher accuracy which would lead to a failure when creating new, previously unseen data.^32^ Therefore, a k-fold cross validation algorithm was applied to all ML algorithms to ensure accuracy and generalizability of the models.

Cross-validation is a resampling procedure that is used to evaluate machine learning models on a limited data sample.^33^ This procedure is often referred to as “k-fold cross-validation.” When a specific value for k is chosen, it can be used in place for k, for example k=10 is a 10-fold cross-validation.^34^ Cross validation is used in machine learning to estimate the skills of a machine learning model in unseen data. Each iteration creates a model with corresponding training and testing dataset, and the final mean accuracy is calculated:

$accuracy_{cv}= \sum_{i=1}^{k} \frac{accuracy_{i}}{k}$ (A.1)

Where *i* denotes the iteration number. The resulting training dataset comprises of approximately *x*% of the data or features from a given subject, and the rest of the (*100-x*)% of the features from each subject is used for validation depending on the number of “folds” (*k*) chosen. This is illustrated in the figure below:


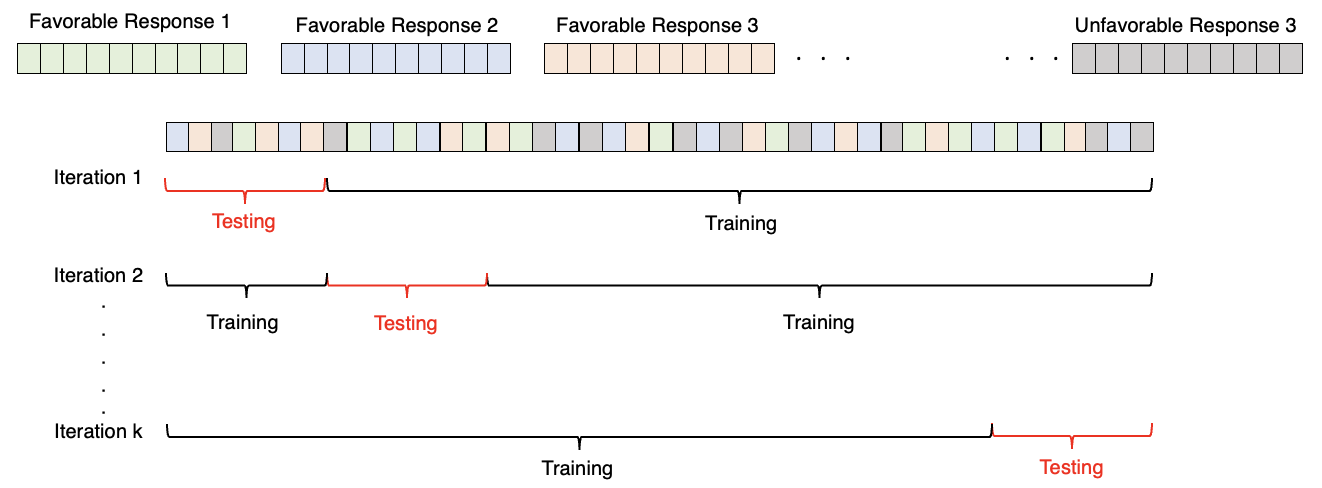


**Figure A1.** K-fold cross validation for favorable and unfavorable neuropsychological outcomes.

This data arrangement helps to evaluate the power of pattern recognition of the algorithms, since a specific part of the data is already seen by the algorithm while training.^35^ The advantages of this is that a limited sample is used in order to estimate how the model is expected to perform in general when used to make inferences on data not used when training the model. It is a popular method because it is simple to understand and requires less biased or less optimistic estimates of the model skill over other methods, such as a train/test split. Most importantly, each observation in the data sample is assigned to the individual group and stays in that group for the duration of the procedure. This gives each sample the opportunity to hold out set 1 time and used to train the model k-1 times. A value of k=10 is very common in the field of applied machine learning.

**Appendix B: Rule-Based Classification**

Rule-based classical ML models were used in this study to build algorithms based on our small-to-medium dataset. They are described in detail below:

1. *Decision Tree*

Decision Trees (DTs) are a non-parametric supervised learning method which have been developed as a divide-and-conquer approach to classification.^36^ DT is built as a tree of nodes within a top-down induction manner which splits the root node consisting of the source data. This splitting is based on certain defined criteria to select the best feature for the split at each node. Some common metrics used include the Gini impurity, information gain, and variance reduction. For both the classic DT method, and our use of DT for feature ranking and KNN, we used the Gini impurity for our conditional metric:

$I_{G}\left( p \right)= \sum_{i=1}^{J} (p_{i}\sum_{k\neq i} p_{k})$ (B.1)

where *p_i_* is the probability of an instance with label *i*, and *J* is the number of classes.^34^ The Gini impurity is defined as the likelihood of incorrectly classifying a random chosen feature if it were randomly labeled according to the distribution of labels within a given subset. It has a lower bound of zero which is obtained when a node consists of data from a single given class. During training of a ML algorithm, the data is split by maximizing the Gini gain which is given by the difference between weighted impurities of the branches from the original impurity.^37^ This process is repeated iteratively until all data within a given node has the same label, or when splitting is no longer adding value to the inferences. DT are easy to interpret since they are prone to overfitting. Figure B1 shows a simple DT. Each node displays the splitting condition, Gini impurity, number of samples in the node and the class assigned to the node.


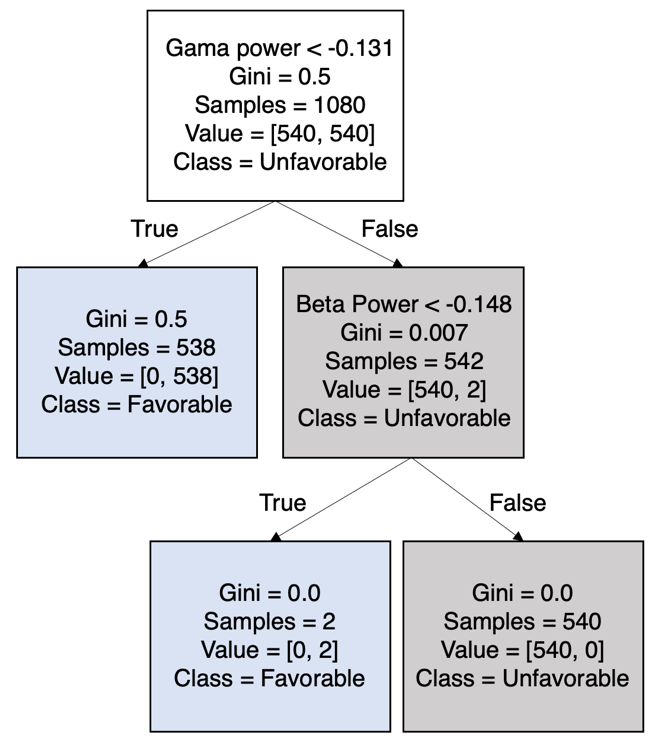


Figure B1. DT method of ranking favorable versus unfavorable outcome.

### Random Forest

Random forests (RFs) are a supervised ML algorithm that builds an ensemble of DT classifiers fit on different sub-samples of the dataset and features.^38^ This method helps in reducing variance of the DT and avoid overfitting by introducing randomization of the data. The output of a RF classification system is an averaged inference of the individual classifiers.^39^ If feature sub-sampling is not performed, the DT may become highly correlated with one another if a certain subset of features is very strongly tied with inference of the target class. In this case, the same subset of features will be used multiple times in developing the DT and make them correlated.

1. *Support Vector Machine*

A support vector machine (SVM) is a supervised learning model which is used for both classification and regression. SVM is accomplished through fitting of a hyper-plane in the N-dimensional space that gives the widest separation between different classes to differentiate between labeled data points. Dimensions of a hyper-plane depends on the number of features that are used.^40^ The distance from the hyper-plane to the nearest data points determines the margin between the two classes. This boundary is determined by a smaller subset of data points, i.e. “support vectors,” and the decision rule is given by:

$\bar{\omega}\cdot\bar{u}+b \geq0$ (B.2)

Where $\bar{\omega}$ is the weight vector which is a vector perpendicular to the hyper-plane, *b* is the intercept term and $\bar{u}$ is the unknown data point. The addition of constraints and variables to calculate $\bar{\omega}$ and *b* turns the equation into:

$\boldsymbol{y}_{\boldsymbol{i}}\left( \bar{\boldsymbol{x}_{\boldsymbol{i}}}\boldsymbol{\cdot}\bar{\boldsymbol{\omega}}\boldsymbol{+b} \right)\boldsymbol{-1=0}$ (B.3)

Where $\bar{x_{i}}$ is the data point and $y_{i}$ is equal to +1 for positive samples and -1 for negative, respectively. Therefore, the constraints of decision rule are equal to either +1 or -1 for positive and negative samples. The margin or width between the decision boundary is given by 2/||$\bar{\omega}$\\ which is to be maximized. Therefore, the formulation of SVM becomes a minimization problem: find $\bar{\omega}$ and *b*, $\frac{1}{2}\bar{w}^{T}\bar{\omega}$ minimized with the constraints specified in the above equation. Lagrange multipliers are used to find the extremum of the function subject to the equally constrained:

$L=\frac{1}{2}\left| |\bar{\omega}| \right|^{2}- \sum\alpha_{i}[y_{i}\left( \bar{w}\cdot\bar{x_{i}}+b \right)-1]$ (B.4)

Where $\alpha_{i}$ are the Lagrange multipliers.^41^ The final decision rule is of the form:

$\sum\alpha_{i}y_{i}\bar{x_{i}}\cdot\bar{u}+b \geq0$ (B.5)

If the data points are not linearly separatable, they are either transformed into another space using different kernels in which they are linearly separable, or a soft margin is used. Some commonly used kernels include a linear, polynomial, and Radial Basis Function (RBF).^42^ If using a soft margin, the cost function to be minimized changes to:

$E\left( \bar{w},b \right)= \left[ \frac{1}{n}\sum_{i=1}^{n} \max(0,1-y_{i}(\bar{w}\cdot\bar{x_{i}}+b)) \right]+ \lambda\left| \left| \bar{w} \right| \right|^{2}$ (B.6)

Where the first term on the right side of the equation corresponds to the hinge loss and the second term is the regularization term.^43^

1. *K-Nearest Neighbor (KNN)*

The k-nearest neighbor (KNN) algorithm is a supervised ML method based on the major assumption that data points from the same classes exist in close proximity with one another. Instead of building a model, KNN stores features of the training dataset during the training phase, and infers the label of the new datapoint from a simple majority vote of “k” (predefined number) of training samples closest to the unknown. If k=1, then the label of the unknown point is assigned based on the closest training sample to it. As the training dataset approaches infinity, the error of one nearest neighbor classifier is upper bounded by twice the Bayes error rate.^44^ The distance metric most commonly used is the Euclidean distance, which measures the distance between two points A and B with coordinates *(x_1_, y_1_)* and *(x_2_, y_2_)* as calculated in the Cartesian plane:

$d\left( A,B \right)= \sqrt{\left( x_{2}-x_{1} \right)^{2}+\left( y_{2}-y_{1} \right)^{2}}$ (B.7)

Since the algorithm is highly dependent on the distance between two points, KNN is highly dependent on the structure of the dataset.^45^ Scaling on the data changes the impact of the assigned label, therefore the optimal value of the number of “neighbors” (k) is highly dependent on the training data. A large value of “k” can reduce overfitting of the data.

### Appendix C: Decision Tree Ranking and Feature Importance

In this section, we describe our methods of feature ranking using decision trees, which were used to select the top six features then used to classify TBI subjects based a K-nearest neighbor’s model.

We let $X=\left[ x_{1},\ldots. x_{S} \right]\in R^{RxS}$ be the diffusion metric for a single region as defined by the regions of the JHU atlas (either DTI, NODDI, or T1) in *R* metric values over *S* subjects. In addition, we have access to the neuropsychological testing labels [*l_1_*,…, *l_S_*] that specify experimental conditions (favorable or unfavorable). We treat each atlas region as a separate datapoint for classification purposes. Feature selection then becomes selecting regions $[d_{1},\ldots, d_{k}]$ whose value exhibit robust generalization.

A Random Forest classifier consists of decision trees for inferring the category label *l* from the DTI, NODDI, or T1 features. Majority voting rule yields the final category through integration of decision trees over all of the trees in the forest. Each tree is trained a random subset of examples (*x_t_, l_t_*).

Let *n_w_* be the total number of examples assigned to a node *w* in the tree and *n_w_^l^* be the number of examples at node w that belong to the category *l*. Let $p_{w}^{l}$ be the empirical frequency of category *l* at node *w*, i.e., $p_{w}^{l}=\frac{n_{w}^{l}}{n_{w}}$. The Gini impurity measures the degree of separation among the classes achieved at a particular node:

$i\left( w \right)= \sum_{l=1}^{L} p_{w}^{l}(1-p_{w}^{l})$ (C.1)

As stated in Appendix B, the *Gini impurity* measures the probability that two independent draws from the multinomial distribution defined by $p_{w}^{l}$are from two different classes.^37^ Each node is associated with a feature *r*  and a threshold value $\eta$. All the examples at node *w* are assigned to one of its two children, w_1_ and w_2_ based on the outcome of the thresholding. We can evaluate the decrease in Gini impurity between node w and its children (w_1_, w_2_):

$\Delta i(w;r,$ $\eta_{v})=i\left( w \right)- \frac{\eta_{w_{1}}}{\eta_{w}}i\left( w_{1} \right)- \frac{\eta_{w_{2}}}{\eta_{w}}i(w_{2})$ (C.2)

During training of the tree, given node *w*, we choose a random subset of features [v_1_,…, v_K_]. We then select a single feature $v^{*}(w)$ and a threshold value $\eta^{*}(w,v^{*})$ that maximizes the reduction in Gini impurity for the node *w*. We repeat this procedure recursively until all leaves of the tree define unique categories.

The Gini importance or *contrast* of feature r is defined as the reduction in the Gini impurity induced by the feature, integrated over all the trees in the forest:

$I_{G}\left( v \right)= \sum_{all trees \{w:r*\left( w \right)=r\}} \sum\Delta i(w;r,$ $\eta^{*}(w,r))$ (C.3)

n this work, we used the Gini importance to rank regions for feature selection. We refer to the value as the *Gini contrast* of a region with regard to the classes in the training set of the decision tree (e.g. different region pathology). The underlying Gini impurity is related to the entropy of the conditional distribution of the labels at node *w*, $i_{e}\left( w \right)= - \sum_{i=1}^{L} p_{w}^{l}\log p_{i}$ by replacing the logarithm log *p_i_* by –(1 - *p_i_*) .^46^ The decrease in Gini impurity quantifies the decrease of labeling uncertainty caused by choosing the feature and threshold. The Gini contrast approximates the expected information gain in the decision tree. It enables selection of voxels that improve the separation among classes at some point of the hierarchical decisions imposed by the decision tree.

We trained a decision tree to rank regions based on Gini contrast. In short, the Gini index is measured by subtracting the sum of squared probabilities of each class from one, in opposite of it, information gain is obtained by multiplying the probability of the class by log (base = 2) of the class probability.^47^ The top 6 features were then selected to be fed into a KNN model to classify the extent of cognitive impairment as measured by the trail making task.

**Appendix D: F1-score measurements of different ML models**

In this section, we evaluate the performance of different ML models utilizing F1-score as the metric. Here we illustrate the mean F1-score of across 5 ML algorithms for trail making A and B in the following table.

|  | Model | AD | FA | MD | ODI | RD | Vic |
| --- | --- | --- | --- | --- | --- | --- | --- |
| Trail A | LR | 0 | 0 | 0 | 0 | 0 | 0 |
| Trail A | DT | 0.353 | 0.448 | 0.404 | 0.43 | 0.244 | 0.267 |
| Trail A | RF | 0.28 | 0.373 | 0.357 | 0.167 | 0.167 | 0.3 |
| Trail A | KNN | 0.317 | 0.397 | 0.26 | 0.383 | 0.387 | 0.233 |
| Trail A | SVM | 0.133 | 0 | 0.2 | 0.067 | 0.133 | 0 |
|  | Model | AD | FA | MD | ODI | RD | Vic |
| Trail B | LR | 0 | 0 | 0 | 0 | 0 | 0 |
| Trail B | DT | 0.397 | 0.377 | 0.352 | 0.554 | 0.333 | 0.273 |
| Trail B | RF | 0.09 | 0.233 | 0 | 0.403 | 0.05 | 0.117 |
| Trail B | KNN | 0.107 | 0.254 | 0.18 | 0.257 | 0.207 | 0.09 |
| Trail B | SVM | 0 | 0 | 0 | 0 | 0 | 0 |
